# Two Visual Training Paradigms Associated with Enhanced Critical Flicker Fusion Threshold

**DOI:** 10.3389/fpsyg.2016.01597

**Published:** 2016-10-26

**Authors:** Tianyou Zhou, Jose E. Náñez, Daniel Zimmerman, Steven R. Holloway, Aaron Seitz

**Affiliations:** ^1^College of Social and Behavioral Sciences, Arizona State University, GlendaleAZ, USA; ^2^Department of Psychology, University of California, Riverside, RiversideCA, USA

**Keywords:** CFFT, visual perceptual learning, reading, ULTIMEYES, directional dot-motion, contrast sensitivity training

## Abstract

Critical flicker fusion thresholds (CFFTs) describe when quick amplitude modulations of a light source become undetectable as the frequency of the modulation increases and are thought to underlie a number of visual processing skills, including reading. Here, we compare the impact of two vision-training approaches, one involving contrast sensitivity training and the other directional dot-motion training, compared to an active control group trained on Sudoku. The three training paradigms were compared on their effectiveness for altering CFFT. Directional dot-motion and contrast sensitivity training resulted in significant improvement in CFFT, while the Sudoku group did not yield significant improvement. This finding indicates that dot-motion and contrast sensitivity training similarly transfer to effect changes in CFFT. The results, combined with prior research linking CFFT to high-order cognitive processes such as reading ability, and studies showing positive impact of both dot-motion and contrast sensitivity training in reading, provide a possible mechanistic link of how these different training approaches impact reading abilities.

## Introduction

The notion that perception is trainable has existed for over a century, and is supported by many "real life" instances, e.g., learning features that distinguish one object or set of objects from another (Gibson, 1953). A more current example of greater ecological value is that while a lay person or novice might fail to detect early tissue changes upon examination of an X-ray scan, expert radiologists can identify low contrast differences in tissue density which might represent precancerous tumors (Snowden et al., 2000). However, a limitation in many perceptual learning studies is that the effects of training are often highly specific to the stimulus features targeted. For instance, Karni and Sagi’s (1991) research documented that visual perceptual learning has three characteristics: (i) local (retinotopic) representation, (ii) orientation-specific asymmetric responding, and (iii) monocular specificity. However, other research suggests that the effect of visual perceptual learning can generalize outside of the trained context. For example, Ahissar and Hochstein (1997) showed that the specificity of visual perceptual learning changes with the difficulty of the tasks (see also Hung and Seitz, 2014). To date there is substantial interest in regard to which perceptual learning can transfer to real world vision tasks and in its potential efficacy as a rehabilitative tool for individuals with visual impairments (Polat, 2009; Deveau and Seitz, 2014).

A key conceptual issue related to perceptual learning serving as a rehabilitative approach is that training fundamental visual processes can lead to broad based changes to vision in general (Deveau et al., 2014). Here, we examine this idea by examining two different visual training approaches; random dot-motion (Gori et al., 2015) and contrast sensitivity training (Deveau et al., 2014) that have been shown to transfer to reading performance. We examine how they impact critical flicker fusion thresholds (CFFTs); a basic visual processing skill (the ability to process a rapidly flickering light until it perceptually fuses into a steady light or perceptually translates from a steady to a flickering light) that has been linked with reading proficiency (Holloway et al., 2013). CFFT was previously thought to be a stable individual-specific function over time, shown to correlate with some cognitive processes related to intelligence (Tanner, 1950; Zlody, 1965) and word decoding ability in reading both real words and meaningless pseudo-words (Holloway et al., 2013). However, CFFT has been shown to be mutable through training on dot-motion tasks (Seitz et al., 2006) and has been hypothesized to be a route to improving reading skills (Holloway et al., 2013; Gori et al., 2015). While correlational, a result showing that both contrast sensitivity training and dot-motion training result in CFFT enhancement would support a model that CFFT improvement is related to the observed reading benefits found through these trainings.

The current study used a publically available contrast sensitivity training task (ULTIMEYES^TM^) and a dot-motion training paradigm to measure each paradigm’s effect on CFFT. It was hypothesized that each training paradigm would result in significant CFFT enhancement. We compared improvement resulting from the two paradigms to see whether one is more effective in producing perceptual learning than the other. These results are compared to an active control group in which participants were exposed to Sudoku, a video game consisting of a cognitive confliction task that has been claimed to improve players’ problem solving ability (Nombela et al., 2011).

## Materials and Methods

### Participants

Participants consisted of 38 healthy university students with normal or corrected to normal visual acuity (20/40 or better on the Snellen scale). In experiment 1, 10 participants trained on ULTIMEYES (4 males, mean age = 20.70, and *SD* = 4.42). In the second experiment, a separate group of ten participants trained on a directional dot-motion task (2 males, mean age = 24.91, *SD* = 8.58). The active control group consisted of a different group of 10 participants trained on Sudoku (5 males, mean = 18.56, and *SD* = 1.01). Participants signed informed consent forms and were compensated with either $75 or 6 class research credits in a psychology course for their participation. All training conformed to the tenants of the Helsinki Declaration for the ethical treatment of human subjects.

### Measurements

Critical flicker fusion threshold was assessed in a dark room (4.7 cd/m^2^) using Maxwellian view presented on a tabletop Macular Pigment Densitometer device (Wooten et al., 1999). The method of limits (the mean of three descending measures from a high to a low frequency of flicker in which the participants reported when the stimulus began to flicker, and three ascending measures from a low frequency to a high frequency in which the participant reported when the flicker stopped) was utilized to measure CFFT. The stimulus consisted of a 1° diameter green (543 nm) round flicking light area against a black background. During testing participants were instructed to sit with their right eye close to the eyehole on the densitometer, in order to fixate their fovea on the green light. The experimenter changed the flicker frequency slowly using a knob, and participants reported when the green light stopped flickering for low-to-high frequency trails, or when the solid green light started flickering for the high-to-low frequency trails. Participants’ flicker frequency was recorded and the average of the six frequencies constituted a participant’s CFFT.

### Visual Perceptual Training

#### Dot-Motion Training

All motion tests and training sessions were run using custom software on computers with 19″ CRT monitors, at a resolution of 1280 × 768, at 75 Hz, controlled by Dell Optiplex Computers running Windows 7 or greater. Participants viewed the displays at a distance of approximately 3 feet. Data collection occurred in a dim room with the ambient light level held constant throughout the experimental sessions.

Motion stimuli consisted of a dynamic array of gray dots (0.2° radius, 400 dots for test, and 300 dots for training) presented on a light gray background with each dot having a 3-frame lifetime for both the testing and training phases.

Motion testing during the pre- and post-test phases of the dot-motion experiment presented dim dynamic motion in four non-cardinal directions (45°, 135°, 225°, and 315°), with display duration of 1000 ms. Following a presentation, a forced-choice display with arrows pointing in the four directions that matched the possible motion directions appeared on the screen. The participants clicked on the arrow that represented the direction in which they believed the dots traveled. The percentage of correct responses was assessed over 5 blocks of 200 trials each, for a total of 1000 trials per test.

In previous research the central task was to remember off-colored (light gray) letters in a set of darker gray letters (Seitz et al., 2006). In the current task, the stimuli were changed to identify brightly colored shape pairs (Holloway, 2016). Each trial started with a pair of target shapes that were displayed for 1000 ms. In 70% of the trials the paired shapes were replaced with a serial presentation of seven randomly generated paired distracter shapes and one pair of target shapes each displayed for 250 ms. In 30% of the trials the paired shapes were replaced with a serial presentation of eight randomly generated paired distracter shapes with no repetition on the target pair. At the end of the serial display participants were required to indicate with a mouse click, whether the target shapes were present. A dim, task-irrelevant dot-motion display was presented in conjunction with either the initial display of the paired shape target or in serial presentation in conjunction with the target pair. This dot-motion was the training stimulus and its direction was constant for each individual participant. This procedure was repeated in 5 blocks of 50 trials each for a total of 250 trials per training session.

#### Contrast Sensitivity Training

For contrast sensitivity training we used ULTIMEYES^TM^, a publically available video-game that trains participants to detect low-contrast Gabor-patches running on a MD510LL/A iPad. At the beginning of each session the participant was shown Gabor patches that varied in contrast level and spatial frequency. The objective was for the participant to identify all the Gabor targets as quickly as possible; a score appeared at the top of the screen, which was determined both by correctly choosing a target and the reaction time for that correct response.

Each training session contained 8–12 exercises for approximately 2 min each. The exercises alternated between static and dynamic types; in the static exercises an array of targets of a single spatial frequency and random orientations was presented at once. In the dynamic exercises, Gabors were presented one at a time, and faded in at random locations on the screen. As training, progressed distracters are added in each trial, changing the task from detection to a discrimination task. Together, these exercises were designed to broadly train visual processes. More detailed aspects of the ULTIMEYES program can be found in Deveau et al. (2014).

#### Sudoku Training

Here we used *Just Sudoku* version 3.3, a free iPad game distributed by Keitgen et al. (2011–2016). We used the classic puzzle with difficulty level is set at “easy”, which consists of a 9 × 9 grid (81 digits total) with 45 digits provided beforehand. The task was to complete the puzzle by selecting the missing digits. The participants were allowed one hint (the correct digit was given in a box) and were not provided with notification of errors. During the game the iPad was locked in a horizontal position to prevent screen rotation. Participants were instructed not to return to the start page prior to completing a puzzle.

### Procedure

On day 1, participants’ visual acuity was assessed using a Snellen scale, prior to beginning the experimental session, to ensure at least 20/40 corrected or non-corrected vision. During this pre-test session, qualified participants were also tested for CFFT on a Macular Pigment Densitometer. This and all other tests were conducted in a dark room without ambient light or sound. One days 2–9, participants were trained on either 25 min of ULTIMEYES, 45 min of dot-motion, or 30 min of Sudoku. The iPads’ screens were cleaned after each session. On day 10, participants completed post-training CFFT tests. Including the pre- and post-training days and the 8 training days, each of the 3 experimental conditions consisted of 10 days a total.

## Results

We first examined performance improvements on the training tasks. In line with findings of previous research, the dot-motion task showed a significant effect of visual perceptual learning; a one-tail *t*-test analysis showed that the percentage of correct responses in identifying dot-motion direction increased from pre-test (*M* = 0.55 and *SD* = 0.14) to post-test (*M* = 0.58 and *SD* = 0.19) [*t*(9) = 1.96, *p* = 0.04, and *r^2^* = 0.30]. A two-tail *t*-test showed that in the Sudoku task, participants’ time for completing one puzzle was significantly reduced from pre-test (*M* = 965.5 and *SD* = 648.09) to post-test (*M* = 276.60 and *SD* = 116.02) [*t*(9) = -3.513, *p* = 0.007, and *r^2^* = 0.58], while the number of errors did not change significantly, showing that their ability for solving the Sudoku puzzle improved. The ULTIMEYES paradigm does not easily allow for pre-test, post-test analysis because the program changes based on individual performance, therefore, analysis of within task performance changes are not discussed.

**FIGURE 1:**
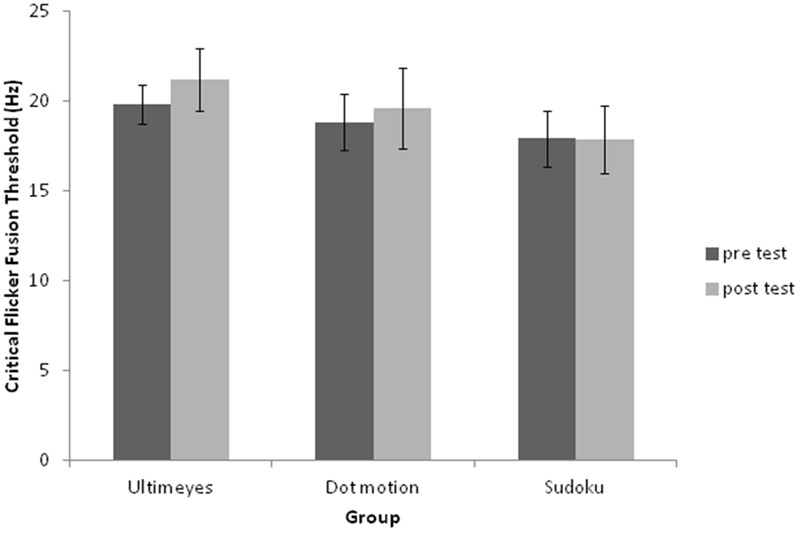
**Critical flicker fusion threshold pre- and post-test performance for the three groups.** The ULTIMEYES and dot-motion groups improved significantly. There was no improvement for the Sudoku group.

The overall results for CFFT were analyzed using a 2 (pre-test vs. post-test) × 3 (groups: ULTIMEYES, dot-motion, and Sudoku) ANOVA. The main effect for pre- and post-test of training for CFFT was significant [*F*(1,27) = 7.82, *p* = 0.009, and *η^2^* = 0.19], with a trend for an interaction for the effect of the experimental condition overall on CFFT [*F*(2,27) = 2.79, *p* = 0.08, and *η^2^* = 0.14]. In accordance with our hypotheses, a paired *t*-test analysis (one-tail) for within group CFFT performance differences showed that the ULTIMEYES post-test score (*M* = 21.20 Hz and *SD* = 1.73) was significantly greater than pre-test (*M* = 19.85 Hz and *SD* = 1.08) [*t*(9) = 2.38, *p* = 0.02, and *r^2^* = 0.39] and likewise CFFT increased for the dot-motion training group; pre-test (*M* = 18.86 Hz and *SD* = 1.56) to post-test (*M* = 19.60 Hz and *SD* = 2.24) [*t*(9) = 2.16, *p* = 0.03, and *r^2^* = 0.34]. Sudoku did not lead to significant pre- vs. post-test performance improvement [*t*(9) = -0.17, *p* = 0.43, and *r*^2^ = 0.003]. The CFFT performance in the three groups is shown in **Table 1** and **Figure 1**.

**Table 1:** Pre- and Post-test CFFT Changes in the three perceptual paradigms.

		Mean (*SD*)	*t*-Score	*p*	*r^2^*
ULTIMEYES	Pre-test	19.85 (1.08)	2.38^∗^	0.02	0.39
	Post-test	21.20 (1.73)			
Dot-motion task	Pre-test	18.86 (1.56)	2.16^∗^	0.03	0.34
	Post-test	19.60 (2.24)			
Sudoku task	Pre-test	17.92 (1.58)	-0.17	0.43	0.003
	Post-test	17.87 (1.90)			

*p-values are from one-tailed *t*-test. ^∗^*p* ≤ 0.05.*

We also tested whether one or more of the groups produced greater CFFT improvement than the others. This was done by calculating pre- vs. post-test difference for each group and comparing difference scores between the groups, using a one-way ANOVA. The *post hoc* analyses showed that CFFT improvement for the ULTIMEYES group (*M* = 1.36 and *SD* = 1.80) was significantly larger than that of the Sudoku control group (*M* = -0.05 and *SD* = 0.96), *p* < 0.05. The CFFT improvement for the dot-motion group (*M* = 0.74 and *SD* = 1.08) was not significantly different from the Sudoku control group (*p* = 0.20) and CFFT improvement of the ULTIMEYES group did not differ from that of the dot-motion group (*p* = 0.31).

## Discussion

Our results show that training on both the dot-motion and contrast sensitivity paradigms resulted in significant increase in CFFT. ULTIMEYES showed a larger, though not statistically significant effect than dot-motion training. On the other hand, the active Sudoku control group did not lead to improvement. These findings support our hypothesis that CFFT improvements are related to perceptual learning.

A key interest for our study was to address whether different perceptual learning-based training approaches that have been implicated in improving reading abilities results in changes to CFFT thresholds. The basis for the hypothesis rests in the magnocellular model of dyslexia (Stein, 2001, 2003). This model links mechanisms of visual motion processing, and temporal processing, such as CFFT with the effective development of reading (Gori et al., 2014; Holloway, 2016). Supporting this model, Gori et al. (2015) conducted multiple studies examining the relationship between dot-motion processing and reading, including showing that dot-motion training led to improvements in reading in participants with dyslexia. Similarly, Polat et al. (2012) and Deveau and Seitz’s (2014) presented evidence that contrast sensitivity training also improved normal people’s reading speed. Our present results showed that both dot-motion training and contrast sensitivity training lead to improved CFFT. Holloway et al. (2013) reported that CFFT is associated with impaired word decoding abilities. Together, such findings help build a mechanistic model suggesting that CFFT may play a role in gating reading abilities. Of note, this model is largely correlational and further research will be required to provide substantiation. Further, while both dot-motion and contrast sensitivity training led to improvements in CFFT, there are numerous differences between the approaches (e.g., stimuli, delivery device, task-design, etc.) and more research will be required to better understand the different impact that each training program may yield in participants.

While the differences in CFFT improvements were not significantly greater after ULTIMEYES training than dot-motion training, our observations revealed a number of attributes inherent in the tasks that would favor use of ULTIMEYES in a rehabilitative setting. First, there was a *time* to completion difference; the former takes approximately 45 min to complete per training session, while completion of an ULTIMEYES session takes about 25 min. Multiplied over the 10-day experimental session this difference represents a considerable time efficiency advantage for ULTIMEYES. Second, there was a boredom issue; during debriefing, dot-motion participants tended to report that it is a somewhat tedious task. This was not a problem reported by ULTIMEYES participants. Third, there was a delivery system issue; ULTIMEYES utilizes a video-game-based technology delivery system presented in an interactive manner on an iPad. It is likely that participants may find this delivery method more ecologically valid than the dot-motion system in its current form, given that video gaming is a common and popular pass time globally.

In sum, the results of current and related research point toward a productive future for utilization of perceptual learning training, e.g., dot-motion and contrast sensitivity, to explore their impact on daily visual tasks. The current study shows that video-based technology, such as that utilized in delivery of the ULTIMEYES paradigm may be especially well-suited for this purpose. Strengthening flicker modulation ability has been shown to be related to increased performance on word decoding, a foundational ability for development of reading. Video-based perceptual learning techniques, show considerable promise as efficient treatment methods for people with developmental dyslexia and other reading problems across the life span. Overall, our results support the hypothesis that findings from basic research conducted within a controlled laboratory setting can be applied to address real-world conditions such as dyslexia and other reading disorders and vision-related problems, such as low acuity and contrast sensitivity.

## Author Contributions

All authors played integral roles in the development and execution of this research project. All authors brainstormed and developed the key components of this study. All authors did the literature review in order to write a well-informed paper. AS developed the Ultimate Eyes training game used in this study. SH Developed the coherent dot motion paradigm we used. TZ, DZ, and JN collected that data. TZ and DZ coded and analyzed the data and JN, AS, and SH reviewed the analyzed data for errors. TZ wrote the original draft of the manuscript, and all other authors edited and co-wrote the manuscript.

## Conflict of Interest Statement

AS is a founder and stakeholder in Carrot Neurotechnology, which developed the ULTIMEYES program described in this presentation. This conflict of interest was reviewed and the research approved by the University of California – Riverside Conflict of Interest Committee and the Human Research Review Board. The other authors declare that the research was conducted in the absence of any commercial or financial relationships that could be construed as a potential conflict of interest.

The reviewer GW and the handling Editor declared their shared affiliation, and the handling Editor states that the process nevertheless met the standards of a fair and objective review.

## References

[B1] Ahissar M. Hochstein S. (1997). Task difficulty and the specificity of perceptual learning. *Nature* 387 401–406. 10.1038/387401a0 9163425

[B2] Deveau J. Lovcik G. Seitz A. R. (2014). Broad-based visual benefits from training with an integrated perceptual-learning video game. *Vision Res.* 99 134–140. 10.1016/j.visres.2013.12.015 24406157 PMC4041814

[B3] Deveau J. Seitz A. R. (2014). Applying perceptual learning to achieve practical changes in vision. *Front. Psychol.* 5:1166 10.3389/fpsyg.2014.01166 PMC4199263 25360128

[B4] Gibson E. J. (1953). Improvement in perceptual judgments as a function of controlled practice or training. *Psychol. Bull.* 50 401–431. 10.1037/h0055517 13112332

[B5] Gori S. Cecchini P. Bigoni A. Molteni M. Facoetti A. (2014). Magnocellular-dorsal pathway and sub-lexical route in developmental dyslexia. *Front. Hum. Neurosci.* 8:460 10.3389/fnhum.2014.00460 PMC4068287 25009484

[B6] Gori S. Seitz A. R. Ronconi L. Franceschini S. Facoetti A. (2015). Multiple causal links between magnocellular–dorsal pathway deficit and developmental dyslexia. *Cereb. Cortex.* 26 4356–4369. 10.1093/cercor/bhv206 PMC6317503 26400914

[B7] Holloway S. R. (2016). *Delineating the “task-irrelevant” perceptual learning paradigm in the context of temporal pairing, auditory pitch, and the reading disabled (Order No. 10107115).* Doctoral Dissertation, Arizona State University Tempe, AZ.

[B8] Holloway S. R. Náñez J. E. Seitz A. R. (2013). Word-decoding as a function of temporal processing in the visual system. *PLoS ONE* 8:e84010 10.1371/journal.pone.0084010 PMC3869845 24376782

[B9] Hung S. C. Seitz A. R. (2014). Prolonged training at threshold promotes robust retinotopic specificity in perceptual learning. *J. Neurosci.* 34 8423–8431. 10.1523/JNEUROSCI.0745-14.2014 24948798 PMC4061387

[B10] Karni A. Sagi D. (1991). Where practice makes perfect in texture discrimination: evidence for primary visual cortex plasticity. *Proc. Natl. Acad. Sci. U. S. A* 88 4966–4970. 10.1073/pnas.88.11.4966 2052578 PMC51788

[B11] Keitgen J. (2011–2016). *Just Sudoku. iTunes.* Available at: https://itunes.apple.com/us/app/just-sudoku-free-to-play-puzzles/id421136193?mt=8 (Accessed: February 28, 2014).

[B12] Nombela C. Bustillo P. J. Castell P. F. Sanchez L. Medina V. Herrero M. T. (2011). Cognitive rehabilitation in Parkinson’s disease: evidence from neuroimaging. *Front. Neurol.* 2:82 10.3389/fneur.2011.00082 PMC3244758 22203816

[B13] Polat U. (2009). Making perceptual learning practical to improve visual functions. *Vision Res.* 49 2566–2573. 10.1016/j.visres.2009.06.005 19520103

[B14] Polat U. Schor C. Tong J. Zomet A. Lev M. Yehezkel O. (2012). Training the brain to overcome the effect of aging on the human eye. *Sci. Rep.* 2:278 10.1038/srep00278 PMC3284862 22363834

[B15] Seitz A. R. Náñez J. E. Holloway S. R. Watanabe T. (2006). Perceptual learning of motion leads to faster flicker perception. *PLoS ONE* 1:e28 10.1371/journal.pone.0000028 PMC1762365 17183655

[B16] Snowden P. T. Davies I. R. L. Roling P. Sowden P. T. Davies I. R. L. Roling P. (2000). Perceptual learning of the detection of features in X-ray images: a functional role for improvements in adults’ visual sensitivity? *J. Exp. Psychol. Hum. Percept. Perform.* 26 379–390. 10.1037/0096-1523.26.1.379 10696624

[B17] Stein J. (2001). The Magnocellular Theory of Developmental Dyslexia. *Dyslexia* 7 12–36. 10.1002/dys.186 11305228

[B18] Stein J. (2003). Visual motion sensitivity and reading. *Neuropsychologia* 41 1785–1793. 10.1016/S0028-3932(03)00179-9 14527541

[B19] Tanner W. P. (1950). A Preliminary Investigation of the Relationship between Visual Fusion of Intermittent Light and Intelligence. *Science* 112 201–203. 10.1126/science.112.2903.201 15442301

[B20] Wooten B. R. Hammond B. R. Land R. I. Snodderly D. M. (1999). A practical method for measuring macular pigment optical density. *Invest. Ophthalmol. Vis. Sci.* 40 2481–2489. 10509640

[B21] Zlody R. L. (1965). The relationship between critical flicker frequency (CFF) and several intellectual measures. *Am. J. Psychol.* 78 596–602. 10.2307/1420921 5839927

